# Effects of Polyethylene Glycol/Porous Silica Form-Stabilized Phase Change Materials on the Performance of Asphalt Binders

**DOI:** 10.3390/ma16155293

**Published:** 2023-07-27

**Authors:** Hao Wang, Gui Pan, Lihong He, Ling Zou

**Affiliations:** 1 School of Civil Engineering, Chongqing Jiaotong University, Chongqing 400074, China; 2 CCCC First Highway Consultants Co., Ltd., Xi’an 710075, China; 3 Yuexiu (China) Transport Infrastructure Investment Ltd., Guangzhou 511458, China; 4 School of Materials Science and Engineering, Chongqing Jiaotong University, Chongqing 400074, China; sunnyhlh@126.com; 5 School of Transportation, Southeast University, Nanjing 211189, China

**Keywords:** asphalt binders, sol–gel method, PEG, road property, temperature-regulating performance

## Abstract

The road performance and temperature-regulating properties of asphalt binders modified with novel polyethylene glycol (PEG)/porous silica (PS) form-stabilized phase-change materials (PEG/PS-fs-PCMs) were studied. PS and PEG were used as the supporting substance and PCMs. The results showed that PEG/PS-fs-PCMs could maintain a maximum weight percentage of 70% without leakage, at temperatures as high as 90 °C. The PEG/PS-fs-PCMs exhibited stable chemical structures, excellent thermal stability, high heat storage density, and suitable phase-change temperature. Based on conventional physical tests, the addition of PEG/PS-fs-PCMs can increase the viscosity and the degree of hardness of asphalt binders; thus, achieving an excellent comprehensive performance of the modified asphalt binder depends on determining the optimal dosage of PEG/PS-fs-PCMs. Additionally, incorporating PEG/PS-fs-PCM particles into the asphalt binder can enhance its ability to withstand permanent deformation at elevated temperatures, while PEG/PS-fs-PCMs mainly act as a filler, weakening the cohesive force of the asphalt molecules, and preventing the ductility of asphalt from expansion, according to DSR and BBR tests. Moreover, the use of PEG/PS-fs-PCMs can enhance the heat transfer properties of the asphalt binders, resulting in an improved temperature regulation performance. However, the accumulation of PEG/PS-fs-PCM particles on asphalt binders can negatively impact the storage stability of the modified asphalt binders, because of the difference in density between the two materials.

## 1. Introduction

Asphalt pavement is a popular choice for road construction, because of its affordability, reliable performance, recyclable properties, excellent wear resistance, and comfortable driving experience [1]. However, asphalt binders are a type of temperature-sensitive material, and their properties are vulnerable to extreme temperature variations, which shortens their lifespan, and increases the cost of road maintenance [2]. Moreover, the black surface of asphalt binders can easily absorb more solar radiation at high temperature, resulting in many pavement and environmental problems, such as asphalt aging, permanent deformation at high temperature, and the UHI effect [3,4]. Numerous methods have been employed by researchers to enhance the durability and functionality of asphalt pavements. The incorporation of modifiers into asphalt binders is widely recognized as an effective method to significantly improve the properties of the binders. Numerous bitumen modifiers, such as nanomaterials [5,6], polymers [7,8], and bio-oil [9] have been reported to date. Among the above modifiers, polymeric materials are widely regarded as the most commonly utilized substances for enhancing the various properties of asphalt binders against most types of distress. Due to their unique nanoscale dimensions, nanomaterials have consistently been shown to significantly enhance the properties of asphalt binders. However, although the aforementioned technologies have been proved to enhance the performance of asphalt pavements to some extent, the thermal damage to asphalt pavements caused by high temperatures occurs, which also exacerbates the urban heat island effect [10,11]. Moreover, these methods are passive, and may not even be representative, given the differences in material, traffic load, and climatic conditions, so they may not be applicable to different asphalt pavements [12,13]. Hence, it is urgent and essential to propose an effective approach to regulate the temperature of, and prevent thermal damage to, asphalt pavements.

Phase-change materials (PCMs) are known for their high efficiency in storing and releasing latent heat, which makes them ideal for maintaining a constant temperature. As a result, they have been used in applications in various fields within heat storage and conversion [14,15,16]. PCMs have been employed in extensive applications in various industries, such as in greenhouses, textiles, solar energy utilization, temperature-regulating fiber manufacturing, medicine, and food packaging [17,18,19]. This characteristic has attracted great interest from a large number of researchers regarding the application of PCMs in thermal regulation in asphalt pavements. Zhang et al. [20] prepared PAC and PACM samples with different PEG contents, and they found that incorporating PCMs in asphalt binders could improve the elastic property of the PAC, store heat energy, and regulate the temperature of asphalt pavements. According to the property of the phase-change thermoregulation agent, Si et al. [21] investigated and compared the thermoregulation of CPCMs at different temperatures, aiming to solve rutting distress in asphalt pavements. Ren et al. [22] synthesized different types of fs-PCMs using the sol–gel method, and the results showed that ACPCMs demonstrate a better stability at a high temperature, and can be applied to asphalt mixture. Zhang et al. [23] employed a vacuum impregnation technique to produce shape-stabilized EG/PEG PCMs, and the aim was to investigate the feasibility of enhancing properties, and controlling the temperature, through this method. Muhammad et al. [24] revealed that the use of suitable PCMs did not adversely affect the rheology of asphalt. Moreover, it was found to be an effective method for regulating temperature variations.

As a solid–liquid transformation PCM, PEG is widely applied in asphalt binders because of its excellent properties, such as its non-toxicity, competitive price, suitable working temperature, and high energy storage density [25,26,27]. However, the original PEG has its inherent disadvantage: liquid leakage in the molten state. Previous research has shown that the addition of PEG in asphalt binders results in a decrease in stiffness, which negatively impacts the high-temperature properties of the asphalt [23,28,29]. To prevent leakage of PEG in practical applications, a promising and effective method is to incorporate PEG into porous materials, to obtain form-stabilized PCMs [30]. In general, there are many types of supporting materials, such as comb-like polymers [31], silicon dioxide [32,33], and white carbon black [34]. In order for fs-PCMs to withstand the high mixing temperature of the asphalt mixture, which typically reaches 185 °C, the supporting materials must have a sufficient thermal stability. Porous silica (PS) is an inorganic porous material that has drawn considerable interest because of its various desirable properties, such as its flame retardancy, commendable thermal stability, and suitable thermal conductivity [35,36]. More importantly, PS contains a large number of porous network structures, and can be utilized as a supporting substance in preparing fs-PCMs, to avoid the leakage of the PEG at high temperatures. Therefore, it is crucial to conduct a thorough investigation into the impact of PEG/PS-fs-PCMs on binder-and temperature-regulating properties.

In this study, PEG and PS were selected as the working materials (PCMs) and support materials, respectively. The heat storage performance, chemical composition and structure, and thermal property of the PEG/PS-fs-PCMs were characterized. On this basis, PEG/PS-fs-PCM-modified asphalt binders were obtained using the melt-blending method, and the physical properties and temperature adjustment performance of the modified asphalt binder were investigated.

## 2. Materials and Methods

Polyethylene glycol (PEG), AR grade, was provided by Jiangsu Haian Petrochemical Plant, Nantong, China; the average molecular weight was 4000 (China). Sodium hydroxide was produced by Changsha Deyue Co., Ltd., Changsha, China. Silica sol was provided by Chongqing Jinglian New Materials Co., Ltd., Chongqing, China. The SK-70 asphalt (KOCH Bitumen Co., Ltd., Chongqing, China) was used for the base asphalt binders, and their performance-testing index is presented in Table 1.

### 2.1. Preparation of PEG/PS-fs-PCMs

**Figure 1:**
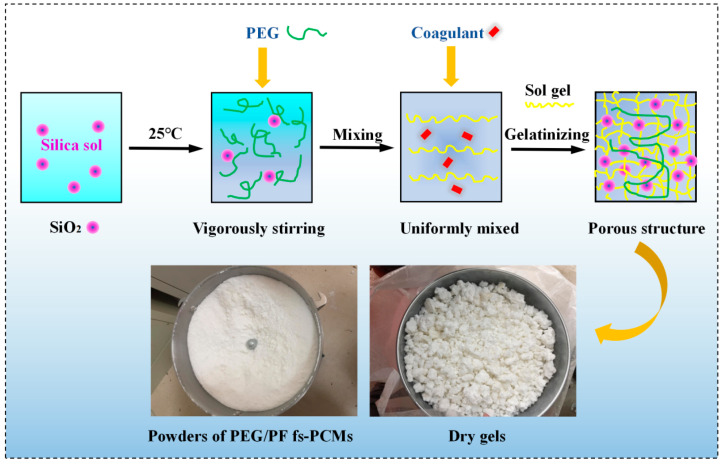
Schematic view of the preparation of PEG/PS-fs-PCMs.

PEG/PS-fs-PCMs were obtained using the sol–gel method, in accordance with the following steps. Firstly, silica sol was mixed with PEG, and stirred vigorously at room temperature, until complete dissolution was achieved. A little sodium hydroxide was then gradually added as an accelerator to the PEG/silica sol mixture, to form the silica sol gel. The mixture was stirred for approximately 10–15 min, until the gelation process was complete. The composites, with the mass ratios of PEG to PS of 9:1, 8:2, 7:3, and 6:4, were tested for leaks, after being kept in an oven at 85 °C for several hours, to determine the maximum ratio of PEG to PS. The test results showed that as long as the impregnation ratio of PEG was equal to, or lower than, that of fs-PCM (70% by weight), the PEG did not leak out from the composite. The fabrication process for the composite material is shown in Figure 1.

### 2.2. Preparation of PEG/PT fs-PCM-Modified Asphalt Binders

This study utilized a high-speed shearing method to modify asphalt binders with PEG/PS-fs-PCMs. The process involved melting the base asphalt at 165 °C, and gradually adding the PEG/PS-fs-PCMs, while stirring at 3000 rpm for 30 min to ensure an even dispersion. Modified asphalt binders with varying PEG/PS-fs-PCM content (0, 5, 10, 15, and 20 weight%) were obtained through this process.

### 2.3. Characterization of Preparation of PEG/PS-fs-PCMs

#### 2.3.1. SEM

The microstructures of the composites were observed using SEM (ZEISS Sigma, Oberkochen, Germany).

#### 2.3.2. DSC

The enthalpy and temperature of the resulting PEG/PS-fs-PCMs were measured using DSC (DSC6000, PE Inc., Long Beach, CA, USA). The experiment was carried out under a nitrogen atmosphere, with a heating rate of 10 °C/min, and a temperature range of 20 °C to 90 °C. Each sample weighed approximately 5–8 mg.

#### 2.3.3. FT-IR

Chemical structures were examined at room temperature using FT-IR (Nicolet iS5, Thermo Fisher, Waltham, MA, USA). All samples were prepared using the KBr plate technique.

#### 2.3.4. TG

The thermal stabilities of the PEG/PS-fs-PCMs at 30–600 °C under a nitrogen atmosphere were evaluated using TGA (TGA 550, New Castle, DE, USA).

#### 2.3.5. Leakage Test

To investigate the leakage resistance of the PCMs, PEG and PEG/PS-fs-PCMs were subjected to an hour-long exposure to 90 °C in an oven. The observation and recording of the process were carried out using a digital camera.

### 2.4. Experimental Methods of the Modified Asphalt Binders

#### 2.4.1. Conventional Properties Test 

The softening point, penetration (15 °C), and ductility (15 °C) of the PEG/PS-fs-PCM-modified asphalt binders were determined, according to the standard methods JTG E20 T0606 [37], JTG E20 T0604 [38], and JTG E20 T0605 [39]. 

#### 2.4.2. Dynamic Shear Rheometer Test (DSR)

The rheological properties of the binders were investigated using a DSR (AASHTO T-315). This test was performed at a of 10 rad/s over a temperature range of 52–76 °C (6 °C/point). 

#### 2.4.3. Bending-Beam Rheometer Test (BBR)

The binders’ ability to resist cracking at low temperatures was evaluated via the BBR test, in accordance with AASHTO T313. At the temperatures of −12 °C and −18 °C, the logarithmic creep rate (m-value) and creep stiffness (S) were measured.

#### 2.4.4. Storage Stability Test

The samples were poured into a glass tube, and this tube was enclosed and placed in a refrigerator for 4 h. Then, the thermal conductivity of the two ends of the samples was calculated, and the thermal conductivity difference between them could be used as an alternative index to characterize the storage stability of the binders.

#### 2.4.5. Temperature-Regulating Test

**Figure 2:**
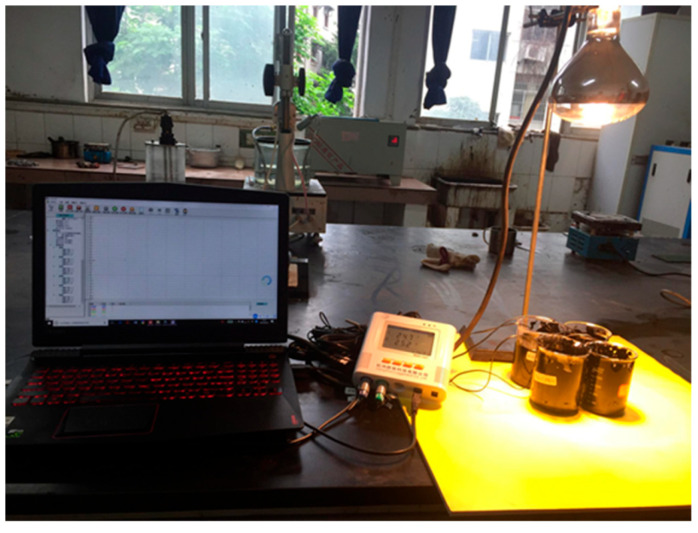
Experimental setup for evaluating the temperature-regulating properties.

For the indoor irradiation experiments, a 400 W solar simulator (QVF133, PHILIPS, Hamburg, Germany) was used as the solar light source. A temperature recorder equipped with thermocouples was used to record the temperature–time curve of the modified asphalt binders, as shown in Figure 2.

## 3. Results

### 3.1. Properties of PEG/PS-fs-PCMs

#### 3.1.1. Morphology Characterization

**Figure 3:**
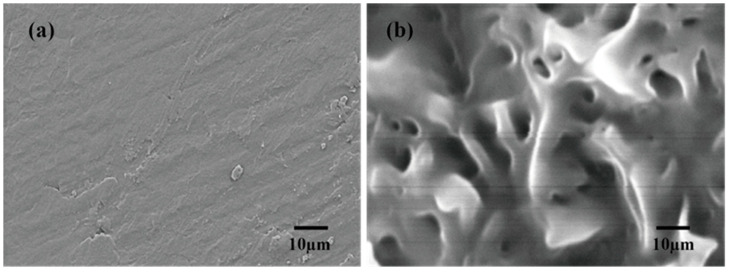
SEM images of the PEG (**a**) and PEG/PS-fs-PCMs (**b**).

As shown in Figure 3, the surface of the PEG appears flat and smooth. As for the composite, it is evident that the PEG molecules are absorbed into the network structure of the PS. Additionally, the pore structure of the silica gel is predominantly occupied by these molecules, with only a few surface pores remaining unfilled. This demonstrates that the silica gel has a highly porous structure, making it an ideal supporting material for PEG. By confining PEG within its porous structure, the silica gel effectively prevents the solid–liquid leakage of PEG at high temperatures. 

#### 3.1.2. Form-Stability Property

**Figure 4:**
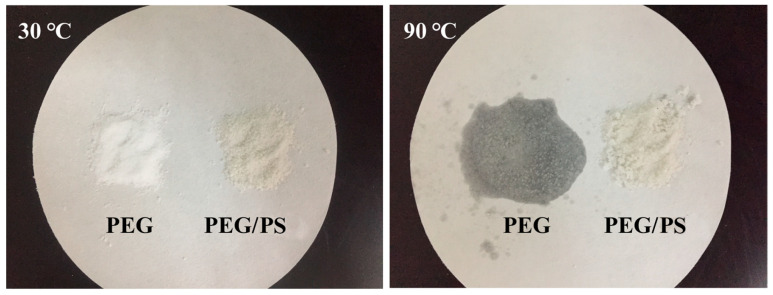
Images of the PEG and PEG/PS-fs-PCMs before and after heating.

Form-stable behavior is a crucial index for fs-PCMs performance. The prepared PEG/PS-fs-PCMs were heated to 90 °C for 1 h, to evaluate their exudation stability. As shown in Figure 4, a clear liquid PEG leaked onto the filter paper after 1 h, implying that its form cannot be retained when the temperature exceeds its melting point. By contrast, PEG/PS-fs-PCMs can retain their original form without any leakage during heating. The results suggest that adding PS to PCMs can improve their form-stability properties, and make them more effective in preventing melt leakage at high temperatures. As a result, the PEG/PS-fs-PCMs prepared in this study are considered to be highly stable when exposed to elevated temperatures.

#### 3.1.3. Chemical Structure

**Figure 5:**
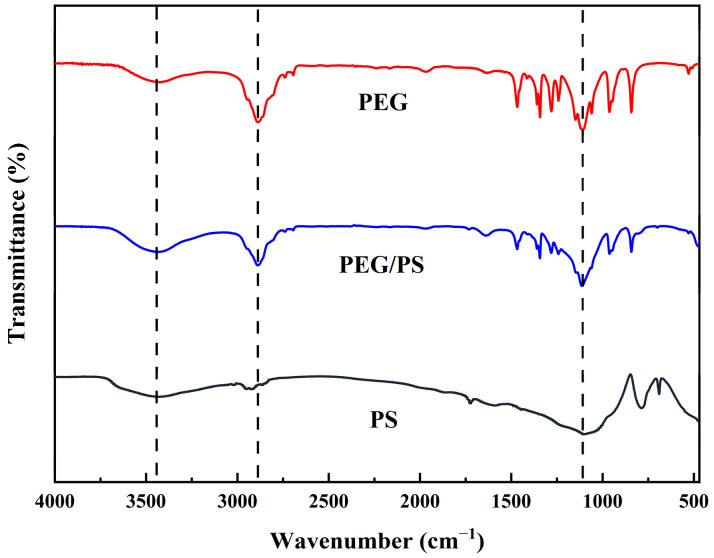
FT-IR spectra of the PEG, PS, and PEG/PS-fs-PCMs.

Figure 5 shows the FT-IR absorption spectra of the samples. The peak at 3432 cm^−1^ in the spectrum of the PEG is caused by the stretching vibration in the OH group. The peak at 1643 cm^−1^ corresponds to the C=O elongation and the asymmetric CO elongation at 1113 cm^−1^. The peaks at 1101, 794, and 468 cm^−1^ in the infrared absorption curve of the PS are typical Si-O-Si peaks. The asymmetric stretching peak of the -OH and the bending vibration of the molecular water are 3444 cm^−1^ and 1732 cm^−1^, respectively, caused by the adsorption of part of the water or capillary pores on the surface of the porous SiO_2_ particles. Obviously, the characteristic peaks of PEG/PS-fs-PCMs are almost the same as those of pure PEG. Furthermore, no new peaks are observed in the composite FT-IR spectrum, indicating that only a physical reaction occurred between the PEG and PS.

#### 3.1.4. Thermal Property

**Figure 6:**
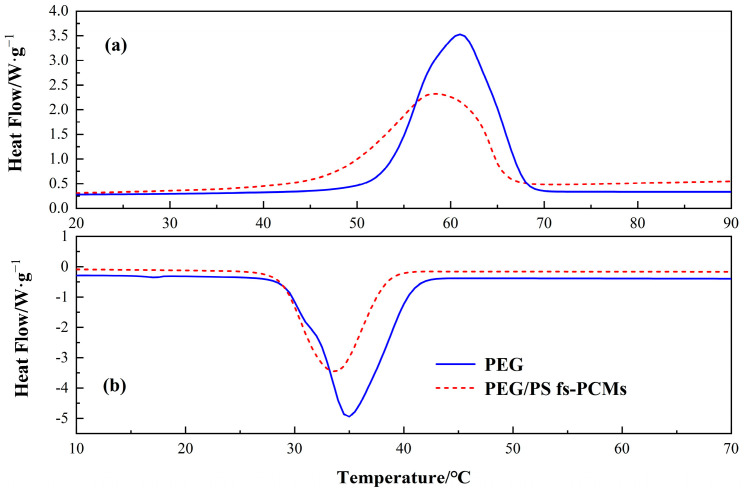
DSC curves of the PEG and PEG/PS-fs-PCMs during heating (**a**) and cooling (**b**) process.

The DSC curves are illustrated in Figure 6. According to the figure, the two samples exhibit similar phase-transition properties, which contain an exothermic peak and endothermic peak when the phase changes. The detailed results of the DSC test are shown in Table 2. As can be seen, the melting and solidification temperatures of the pure PEG were 61.2 °C and 34.9 °C, respectively, and those of the PEG/PS-fs-PCMs were 61.5 °C and 33.8 °C, respectively. Notably, the melting temperature of the PEG/PS-fs-PCMs is quite close to that of the PEG, but the solidification temperature decreases obviously after the addition of the PS, which is possibly caused by the volume restriction effect of the porous structure network of the PS [31].

Table 2 shows that the fusion and solidification latent heat for the pure PEG are calculated to be 182.4 J/g and 171.3 J/g, respectively. On the other hand, the PEG/PS-fs-PCMs have a fusion and solidification latent heat of 121.6 J/g and 114.7 J/g, respectively. Obviously, the latent heat of the PEG/PS-fs-PCMs slightly declines when compared to the PEG. The decrease in the transition enthalpy is primarily caused by a reduction in the mass percentage of the PEG after the incorporation of the PS. PEG is important for thermal energy storage during melting or solidification. Despite this reduction, the latent heat of the PEG/PS-fs-PCMs is higher compared to previous fs-PCMs studied by other researchers. Moreover, the phase-transition temperatures of the PEG/PS-fs-PCMs are similar to the deformation temperatures of asphalt pavements during summer. Therefore, the DSC test results demonstrate that PEG/PS-fs-PCMs have a desirable heat storage density, and favorable phase-transition temperatures, making them a practical option for use in hot mixed asphalt.

#### 3.1.5. Thermal Stability of PEG/PS-fs-PCMs

**Figure 7:**
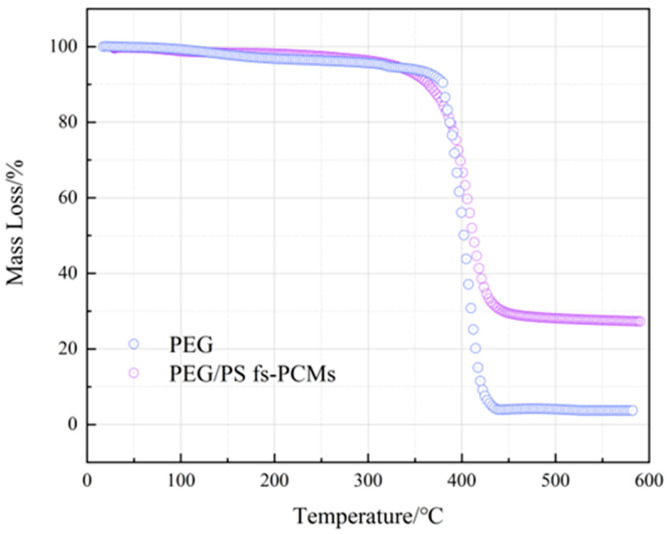
TGA curves of the PEG and PEG/PS-fs-PCMs.

To investigate the ability of PEG/PS-fs-PCMs to withstand high temperatures in asphalt mixtures, the thermal stability properties of the samples were analyzed using TGA. The results are presented in Figure 7, which shows the thermogram of the pristine PEG displaying an onset of weight loss at approximately 311.2 °C. Additionally, there is a significant decline observed from 388.4 to 407.8 °C, with a weight loss of 96.7%. This weight loss corresponds to the evaporation and decomposition of the PEG. Otherwise, the PEG/PS-fs-PCMs exhibit a relatively stable TG curve, and both the initial degradation temperature (Ton) and the temperature of the maximum decomposition rate (Tmax) are a little higher than those of the PEG. Specifically, the Ton and Tmax of the PEG/PS-fs-PCMs reach 314.5 °C and 410.9 °C, respectively, indicating that the dimensional pore structures of the PS have a positive effect on the thermal stability of the composites. Moreover, the amount of residual PEG/PS-fs-PCMs in the composite depends on the loading amount of PS, and the quantity of PEG impurities present. The results of the TG analysis indicate that the PEG/PS-fs-PCMs have a sufficient thermal stability, and can be utilized as a thermoregulating agent in asphalt at a mixing temperature of up to 185 °C.

### 3.2. Performance of PEG/PS-fs-PCM-Modified Asphalt Binders

#### 3.2.1. Conventional Physical Performance

**Figure 8:**
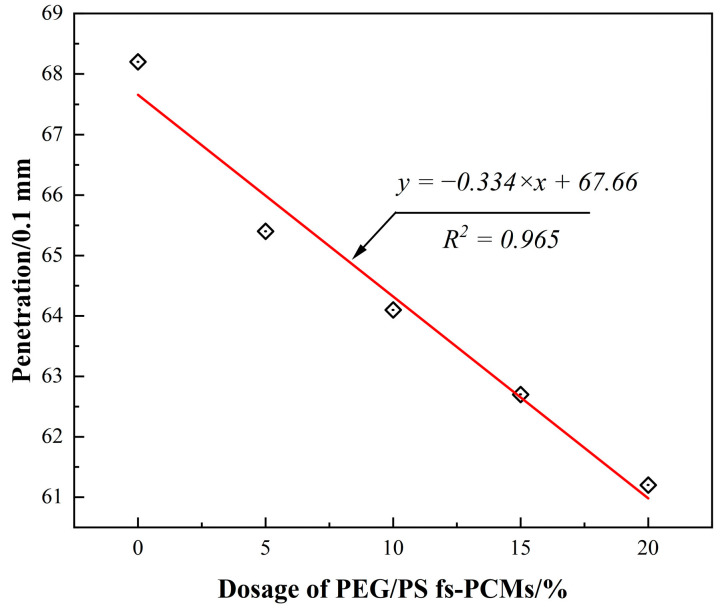
Correlation between the dosage of PEG/PS-fs-PCMs and the penetration of asphalt binders.

Generally, the permeability value is widely used to evaluate the degree of hardness of asphalt at intermediate temperatures. A low permeability value reflects that the asphalt is hard and stiff. Figure 8 shows the results of the permeability test. As can be seen, the permeability values of all the samples decreased almost linearly with the increase in PEG/PS-fs-PCMs content. The linear fitting is convincible, because R^2^ is higher than 0.95. Specifically, the permeability value reduces by 6.1% compared with the control sample after 10% PEG/PS-fs-PCMs are added into virgin asphalt, and when the dosage of PEG/PS-fs-PCMs is increased to 20%, the permeability value is reduced by 10.3%. The obtained results reveal that the addition of the PEG/PS-fs-PCMs can improve the stiffness rutting resistance of asphalt binders. This may be explained by the fact that the oily phase can be absorbed by PEG/PS-fs-PCMs particles, thus increasing the asphaltene component of the asphalt, and restricting the flow of the asphalt, thereby enhancing its stiffness. 

**Figure 9:**
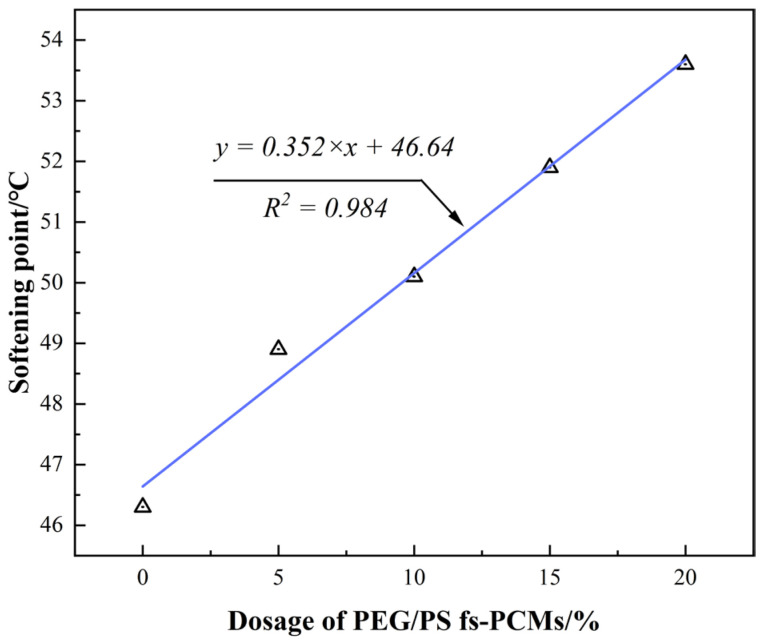
Correlation between the dosage of PEG/PS-fs-PCMs and softening point of asphalt binders.

The softening point is a crucial parameter that indicates the stability of asphalt binders under high service temperatures. In general, a higher softening point indicates a better high-temperature stability in asphalt binders. According to Figure 9, the softening points linearly increase with the increase in the PEG/PS-fs-PCMs dosage, which is different from the permeability value. For example, the softening point of the modified asphalt with the modifier dosage of 5%, 10%, 15%, and 20% increases up to 5.6%, 8.2%, 12.1%, and 15.8%, respectively, when compared with that of the virgin asphalt, thus proving that the PEG/PS-fs-PCMs enhance the high-temperature property of asphalt. Hence, it is well supported that the viscosity and the degree of hardness of asphalt binders can be increased by adding PEG/PS-fs-PCMs. As a result, the asphalt binders become more compact and stable, thus eventually enhancing their high-temperature stability.

**Figure 10:**
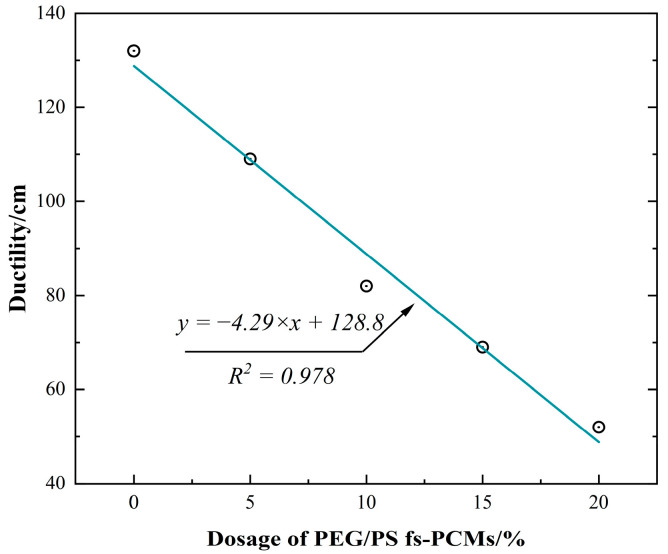
Correlation between the dosage of PEG/PS-fs-PCMs and the ductility of asphalt binders.

The ductility is a crucial factor in evaluating the low-temperature performance. It is important to note that a higher ductility value indicates a poorer low-temperature performance. The test results for the ductility at 15 °C are shown in Figure 10. It is obvious that the ductility value is reduced after PEG/PS-fs-PCMs are added. Specifically, when the dosages of PEG/PS-fs-PCMs are 5%, 10%, 15%, and 20%, respectively, the asphalt binder ductility values decrease to 17.4%, 36.5%, 47.8%, and 60.6%, respectively, indicating that PEG/PS-fs-PCMs negatively affect the asphalt low-temperature performance, due to an increased hardness and stiffness. In conclusion, the results of conventional physical tests show that the optimal dosage of PEG/PS-fs-PCMs is the key factor in obtaining a modified asphalt binder with an excellent comprehensive performance.

#### 3.2.2. High-Temperature Performance

The dynamic shear rheometer (DSR) is a commonly employed technique to assess the ability of asphalt binders to withstand permanent deformation at high temperatures. The rutting resistance index (G*/sinδ) is an important parameter that is strongly correlated with the rutting behavior of asphalt binders. Higher values of G*/sinδ indicate greater resistance to permanent deformation. This study examines the impact of various dosages of PEG/PS-fs-PCMs on the rheological characteristics of asphalt binders.

**Figure 11:**
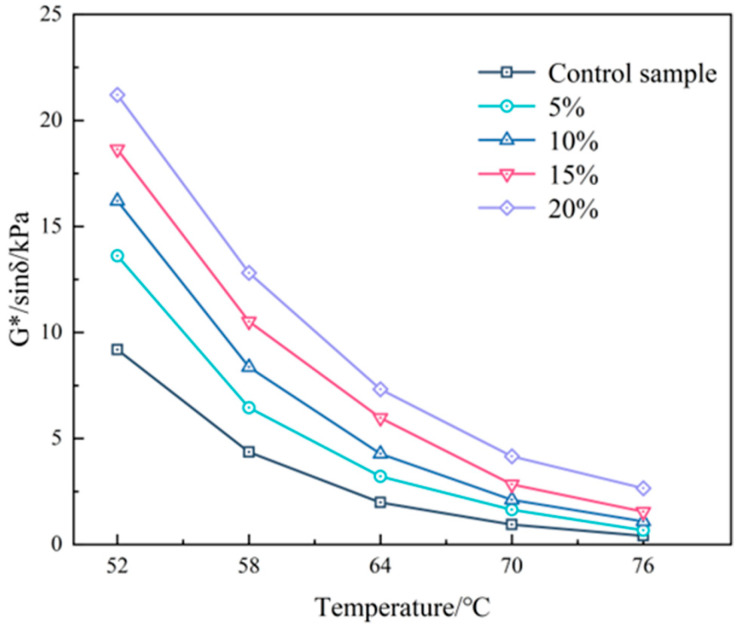
Rutting resistance of the PEG/PS-fs-PCM-modified asphalt binders.

As Figure 11 indicates, the rutting factor G*/sinδ of all the samples exhibits an apparent downward trend as temperatures increase, implying the typical temperature-dependency properties of asphalt. Moreover, the G*/sinδ obviously increases as the content of PEG/PS-fs-PCMs increases, indicating that the high-temperature anti-rutting performance can be greatly improved by adding PEG/PS-fs-PCMs. Specifically, compared to virgin asphalt, the increased amplitude of G*/sinδ in the PEG/PS-fs-PCM-modified asphalt with the contents of 10% and 20% is the greatest at 64 °C, at 52.3% and 125.7%, respectively. This can be explained as follows. On one hand, the PEG/PS-fs-PCMs particles with a greater surface area can adsorb light components in the asphalt, thus enhancing its anti-rutting property. On the other hand, some heat energy can be absorbed by the PEG/PS-fs-PCMs particles during the heating process; thus, the asphalt binder faces a lower actual test temperature. Moreover, the high-temperature properties of the asphalt binder are also enhanced due to the presence of silica, as demonstrated in other studies.

**Figure 12:**
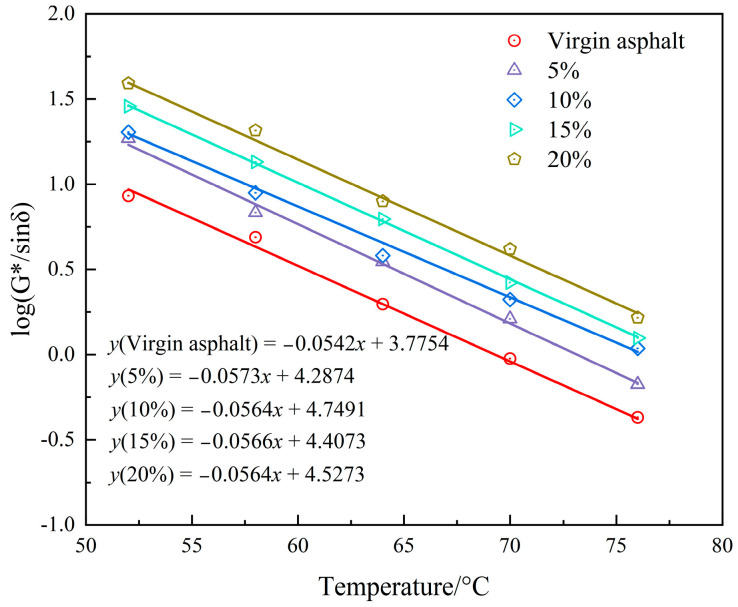
Correlation between the log(G*/sinδ) and temperatures of the modified asphalt binders.

Furthermore, the failure temperatures of the samples can be determined by calculating the linear fitting curves of log(G*/sinδ) at a G*/sinδ value of 1.0 KPa. Figure 12 and Table 3 reveal the failure temperature of the PEG/PS-fs-PCM-modified asphalt. The failure temperature of the control sample is about 68.1 °C. When the content of the loaded PEG/PS-fs-PCMs increases from 0% to 20%, however, the failure temperature of the modified asphalt increases from 68.1 °C to 82.2 °C, resulting in a 20.7% growth rate compared to the virgin asphalt. This suggests that the rutting resistance has improved.

#### 3.2.3. Low-Temperature Performance

To examine the impact of PEG/PS-fs-PCMs on the low-temperature rheological characteristics of binders, we conducted a BBR test, and measured the creep rate (m) and stiffness modulus (S) as the evaluation criteria. Generally speaking, a lower creep stiffness indicates less tensile stress, and a higher creep rate indicates more stress relaxation. Therefore, the asphalt binders with a larger creep rate and/or smaller creep-stiffness modulus are believed to exhibit a better low-temperature performance. 

**Figure 13:**
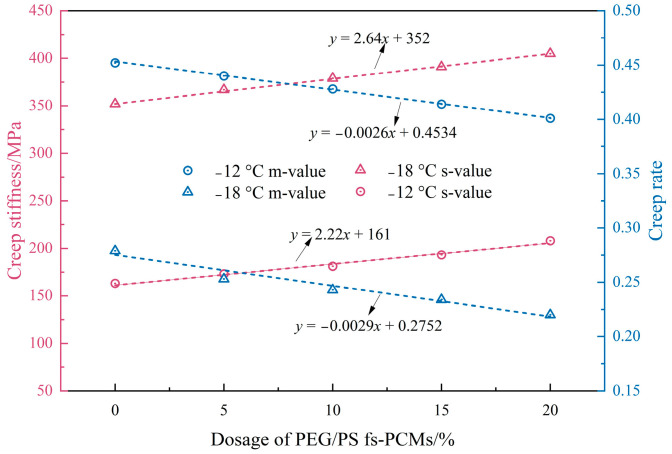
BBR test results for the asphalt samples.

As observed from Figure 13, the creep-rate value of all the PEG/PS-fs-PCM-modified asphalt binders decreases, while the creep stiffness modulus value increases, as the temperature decreases, indicating that the temperature dependency of the asphalt binders does not change after PEG/PS-fs-PCMs are added. However, the addition of PEG/PS-fs-PCMs to asphalt binders results in an increase in S values, and a slight decrease in the creep rate. This indicates that the rheological performance of the binders at low temperatures is reduced, making them more susceptible to cracking. This is possibly because the PEG/PS-fs-PCMs mainly act as filler, and weaken the cohesive forces of the asphalt molecules, thus preventing the ductility extension of the asphalt. According to the Superpave standard, the asphalt binder’s S-value should be lower than 300 MPa, and the m-value should not exceed 0.3. It is impressive that all the samples meet the specified requirements for the m-values and S-values. Therefore, it is crucial to control the dosage of PEG/PS-fs-PCMs within 20%, to avoid further degradation of the low-temperature performance. 

#### 3.2.4. Compatibility Stability Performance

**Figure 14:**
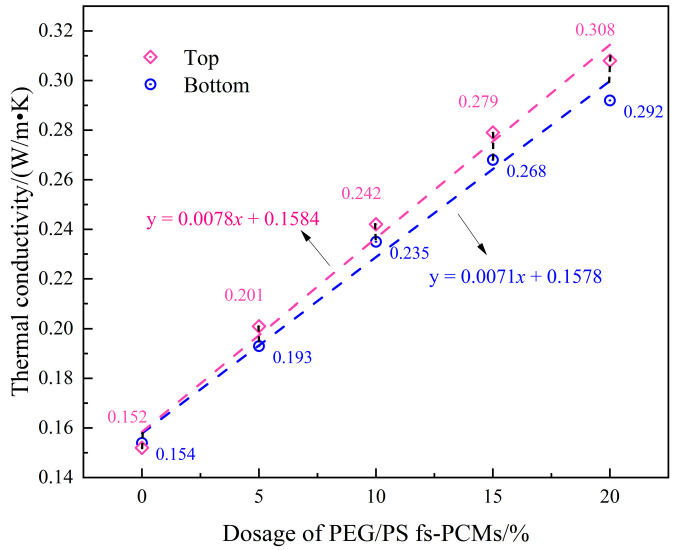
Storage stability of the PEG/PS-fs-PCM-modified asphalt binders.

It is universally known that modified asphalt binders are multiphase systems, and there is a difference in the density between the asphalt and the modifying agent. The phase separation and performance inhomogeneity of binders during high-temperature storing and pumping are significantly influenced by the compatibility stability. This can be observed in the results of the compatibility stability test, as shown in Figure 14. 

It is observed that the thermal conductivity of the top and bottom parts of the virgin asphalt binder is relatively stable, with values of 0.154 and 0.152 W/m · K, respectively, indicating that the virgin asphalt is in a stable storage state. The addition of PEG/PS-fs-PCMs to modified asphalt binders results in an increase in thermal conductivity, indicating an improvement in the heat transfer performance. It is important to note that the difference in thermal conductivity between samples becomes more pronounced as the dosage of PEG/PS-fs-PCMs increases. For example, when the content of PEG/PS-fs-PCMs is 10% and 20%, the difference in the thermal conductivity of the modified asphalt binders increases by 0.007 and 0.016 W/m · K, respectively. This is probably because a small dosage of PEG/PS-fs-PCMs is aggregated on the top of the samples, due to the density difference. The results suggest that a high concentration of PEG/PS-fs-PCMs has an adverse effect on the storage stability of asphalt binders. Therefore, to achieve a modified asphalt with an exceptional performance, it is crucial to determine the optimal amount of PEG/PS-fs-PCM modifier, in conjunction with the ductility test and BBR test.

#### 3.2.5. Temperature-Regulating Performance

**Figure 15:**
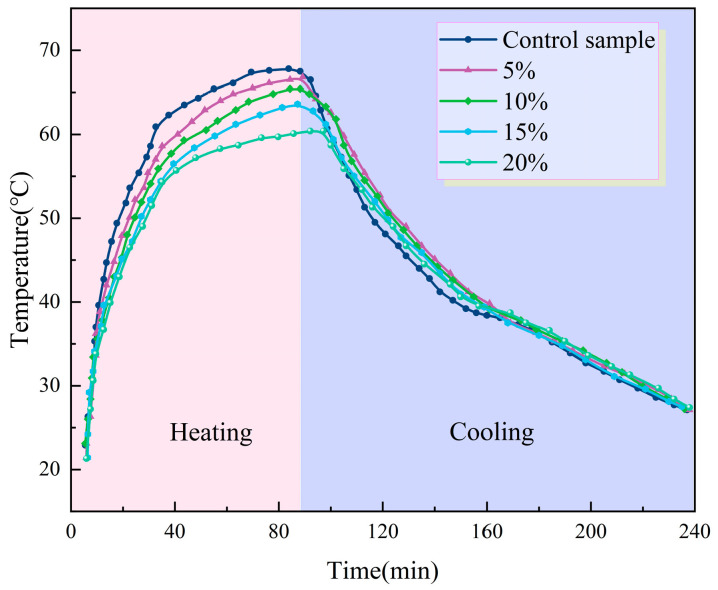
Temperature curves of the PEG/PS-fs-PCM-modified asphalt binders.

To more intuitively characterize the impact of the prepared samples on the temperature-regulating performance of asphalt binders, an indoor irradiation test was conducted in the laboratory. Figure 15 depicts the temperature–time profiles of both the virgin asphalt and the PEG/PS-fs-PCM-modified asphalt binders during the irradiation process. The figure shows that the heating rate of the modified asphalt is initially similar to that of the virgin asphalt under light irradiation. As the irradiation time increases, and the temperature reaches the fusion point of the PEG/PS-fs-PCMs, an inflection point appears in the temperature variation curves, indicating a phase transition, and the absorption of thermal energy. Consequently, the temperature of the modified asphalt binders increases at a slower rate. Meanwhile, the thermal-regulating performance of the samples is related to the incorporated content of PEG/PS-fs-PCMs. As the dosage of PEG/PS-fs-PCMs increases, the thermal-regulating performance becomes better and better. The difference in the maximum peak temperature between the virgin asphalt and the 20% modified asphalt binders reaches 6.4 °C at the end of irradiation. Therefore, PEG/PS-fs-PCMs have the ability to absorb thermal energy, and control the temperature of the hot mix asphalt while it is being heated.

## 4. Conclusions

In this work, the experimental method used in the study contributes to a better knowledge of the effects of the prepared PEG/PS-fs-PCMs on the performance of the asphalt binder. From the experimental results and analyses, the following conclusions can be summarized.

(1) The prepared PEG/PS-fs-PCMs exhibit excellent thermal stability, commendable heat storage density, and appropriate phase-change temperatures for use as a thermoregulation modifier of an asphalt binder, as demonstrated via FT-IR, DSC, and TG experiments.
(2) Incorporating PEG/PS-fs-PCMs into the asphalt binder results in a decrease in the penetration and ductility, and an increase in the softening point. This may be attributed to the addition of PEG/PS-fs-PCMs weakening the cohesive force of the asphalt molecules, and limiting the ductility of the asphalt.
(3) The rutting resistance index (G*/sinδ) of the asphalt binder improves with the increasing content of PEG/PS-fs-PCMs, and so does the failure temperature, suggesting that the addition of the fs-PCMs is beneficial in enhancing the rutting resistance of the asphalt binder at high temperatures.
(4) The creep rate of the asphalt binder decreases, while the creep stiffness increases, due to the incorporation of PEG/PS-fs-PCMs, resulting in worse low-temperature properties. Therefore, it is not recommended to further increase the dosage of fs-PCMs.
(5) Temperature regulation tests demonstrate that the addition of PEG/PS-fs-PCMs induces significant hysteresis in the asphalt binder temperature profiles, implying the outstanding temperature regulation capability of the fs-PCMs. The modified asphalt binder shows a maximum temperature difference of approximately 6.4 °C, compared to the virgin asphalt.
(6) Overall, considering the high-temperature performance and excellent thermoregulation efficiency, the appropriate dosage may be determined to be 10%. Furthermore, an aging test and workability analysis will be performed in the future, for a more comprehensive evaluation of the performance of the PEG/PS-fs-PCM-modified asphalt binder.

## Figures and Tables

**Table 1:** Technical testing index of the base asphalt.

Test Indices	Technical Requirements	Measured Values	Specification
Softening point (°C)	≥46	48.1	T0606-2011
Dynamic viscosity (60 °C Pa s)	≥180	223	T0620-2011
Ductility (10 °C cm)	≥25	37	T0605-2011
Penetration (25 °C mm)	60–80	66.3	T0604-2011

**Table 2:** Data for the DSC curves of PEG and PEG/PS-fs-PCMs.

Samples	Tm (°C)	ΔHm (J/g)	Tc (°C)	ΔHc (J/g)
PEG	61.2	182.4	34.9	171.3
PEG/PS-fs-PCMs	61.5	121.6	33.8	114.7

**Table 3:** Failure temperature of PEG/PS-fs-PCM-modified asphalt binders.

Samples	Control Sample	5%	10%	15%	20%
R^2^	0.975	0.964	0.951	0.972	0.968
Failure temperature	68.1 °C	73.2 °C	77.4 °C	78.1 °C	82.2 °C

## Data Availability

Data sharing not applicable.
